# [^18^F]DPA-714 PET Imaging Reveals Global Neuroinflammation in Zika Virus-Infected Mice

**DOI:** 10.1007/s11307-017-1118-2

**Published:** 2017-09-12

**Authors:** Kyle Kuszpit, Bradley S. Hollidge, Xiankun Zeng, Robert G. Stafford, Sharon Daye, Xiang Zhang, Falguni Basuli, Joseph W. Golden, Rolf E. Swenson, Darci R. Smith, Thomas M. Bocan

**Affiliations:** 1 Molecular and Translational Sciences Division, U.S. Army Medical Research Institute of Infectious Diseases, 1425, Porter St., Ft. Detrick, Frederick, MD 21702 USA; 2 Virology Division, U.S. Army Medical Research Institute of Infectious Diseases, 1425, Porter St., Ft. Detrick, Frederick, MD 21702 USA; 3 Pathology Division, U.S. Army Medical Research Institute of Infectious Diseases, 1425, Porter St., Ft. Detrick, Frederick, MD 21702 USA; 4 Imaging Probe Development Center, National Heart, Lung, and Blood Institute, National Institutes of Health, 9800 Medical Center Drive, Bldg. B., #2034, Bethesda, MD 20892 USA

**Keywords:** Zika virus, Animal model, Mice, Pathology, Neuroinflammation, TSPO, DPA-714, PET imaging, Therapeutics

## Abstract

**Purpose:**

The association of Zika virus (ZIKV) infection and development of neurological sequelae require a better understanding of the pathogenic mechanisms causing severe disease. The purpose of this study was to evaluate the ability and sensitivity of positron emission tomography (PET) imaging using [^18^F]DPA-714, a translocator protein (TSPO) 18 kDa radioligand, to detect and quantify neuroinflammation in ZIKV-infected mice.

**Procedures:**

We assessed ZIKV-induced pathogenesis in wild-type C57BL/6 mice administered an antibody to inhibit type I interferon (IFN) signaling. [^18^F]DPA-714 PET imaging was performed on days 3, 6, and 10 post-infection (PI), and tissues were subsequently processed for histological evaluation, quantification of microgliosis, and detection of viral RNA by *in situ* hybridization (ISH).

**Results:**

In susceptible ZIKV-infected mice, viral titers in the brain increased from days 3 to 10 PI. Over this span, these mice showed a two- to sixfold increase in global brain neuroinflammation using [^18^F]DPA-714 PET imaging despite limited, regional detection of viral RNA. No measurable increase in ionized calcium binding adaptor molecule 1 (Iba-1) expression was noted at day 3 PI; however, there was a modest increase at day 6 PI and an approximately significant fourfold increase in Iba-1 expression at day 10 PI in the susceptible ZIKV-infected group relative to controls.

**Conclusions:**

The results of the current study demonstrate that global neuroinflammation plays a significant role in the progression of ZIKV infection and that [^18^F]DPA-714 PET imaging is a sensitive tool relative to histology for the detection of neuroinflammation. [^18^F]DPA-714 PET imaging may be useful in dynamically characterizing the pathology associated with neurotropic viruses and the evaluation of therapeutics being developed for treatment of infectious diseases.

**Electronic supplementary material:**

The online version of this article (10.1007/s11307-017-1118-2) contains supplementary material, which is available to authorized users.

## Introduction

ZIKV is a human pathogenic arthropod-borne flavivirus which is similar to dengue, West Nile, Japanese encephalitis, and yellow fever viruses [1, 2]. ZIKV is typically asymptomatic and mortality is rare. However, neurological complications resulting in Guillain-Barré syndrome (GBS) [3, 4], encephalopathy [5], meningoencephalitis [6], and acute myelitis [7] occurs in some adults. ZIKV is becoming a major global public health concern because infection in pregnant women is linked to congenital abnormalities including microcephaly, spontaneous abortion, and intrauterine growth restriction [8]. Many questions remain unanswered about the mechanisms by which ZIKV might cause severe neurological sequelae including congenital defects.

*In vivo* molecular imaging has been used to characterize disease progression and evaluate drugs in the areas of neuroscience, cardiovascular, inflammation, and oncology, but application of imaging to infectious diseases has been limited (reviewed in [9]). More specifically, application of *in vivo* imaging in evaluation of animal models of ZIKV infection has not been described. The power of molecular imaging lies in its ability to provide a non-invasive, spatiotemporal measurement of pathogen infection and its effects on key biological processes such as metabolism and inflammation. Positron emission tomography (PET) imaging using 2-deoxy-2-[^18^F]fluoro-d-glucose ([^18^F]FDG) has been commonly used as a radiotracer in clinical and basic research. [^18^F]FDG is an analog of glucose that accumulates preferentially in cells based on their metabolic activity rather than their cell type and has been used to assess tissue metabolism. In general, [^18^F]FDG has been used substantially to assess inflammation. As key components of the inflammatory response, infiltrating inflammatory cells utilize glucose at a much higher rate than peripheral non-inflammatory cells [10]. Therefore, the increased glucose metabolism of these inflammatory cells has become an important and frequently used target in PET imaging of inflammation. [^18^F]FDG uptake, however, is not cell-type specific, making it sometimes difficult to differentiate signal derived from an active inflammatory response from signal originating from hypermetabolic cells or tissues *not* associated with the local inflammatory response (*e.g.*, brain or muscle). In contrast, the radiotracer [^18^F]DPA-714 monitors the surface molecule TSPO [11] which is highly upregulated in central nervous system (CNS) activated microglia, macrophages, neutrophils, lymphocytes, and reactive astrocytes [12–19] and has therefore emerged as a promising target for the dynamic analysis of neuroinflammation using PET imaging.

Immunocompetent adult mice infected with ZIKV can develop a transient viremia but do not demonstrate signs of morbidity or mortality. In contrast, knockout mice deficient in the type I or II interferon (IFN) response were found to be highly susceptible to ZIKV infection where virus replicated in multiple organs including the brain [20–23]. To assess neuroinflammation associated with ZIKV infection, [^18^F]DPA-714 PET imaging was performed in a recently described murine model of ZIKV infection where the type I IFN response is transiently suppressed. This model utilizes wild-type C57BL/6 mice treated with a non-cell depleting monoclonal antibody (MAb-5A3) to disrupt type I IFN signaling [24]. Mice infected by intraperitoneal inoculation resulted in 100 % mortality and animals experienced weight loss, viremia, hind-limb paralysis, and severe neuropathology. CNS pathology is a prominent feature of the animal model. Encephalitis/encephalomyelitis, characterized by neuronal death, astrogliosis, microgliosis, scattered necrotic cellular debris, and inflammatory cell infiltrates, are observed in the brain lesions [24]. Given that an intact immune system exists in this model, blockade of the IFN I system upon infection allows for an immune response to be elicited. Evaluation of countermeasures to ZIKV infection in this model may more closely mimic the human response.

In the current study, ZIKV-infected mice administered MAb-5A3 (5A3) antibodies to inhibit IFN signaling were evaluated at early, middle, and late stages of infection by PET imaging using [^18^F]DPA-714. [^18^F]DPA-714 was selected for use due to its longer isotope half-life, high affinity, lower non-specific binding, and increased bioavailability to the brain. To control for effects on neuroinflammation stemming from 5A3 activity or ZIKV alone, 5-week-old female C57BL/6 mice were separated into three treatment groups: (1) phosphate-buffered saline (PBS) + 5A3, (2) ZIKV + PBS, and (3) ZIKV + 5A3. Individual cohorts of mice from each group were evaluated for changes in neuroinflammation at days 3, 6, and 10 post-infection (PI). Histologic analysis of tissue specimens was performed to correlate the imaging findings with the classical means of assessing the neuropathology owing to ZIKV infection. Unlike a histologic assessment, imaging approaches have the potential to dynamically characterize in a live animal model of ZIKV infection the disease process in a functional context. This approach can be applied across a broad spectrum of viruses and can be used to evaluate drug therapy both preclinically and clinically.

## Materials and Methods

### Virus

The Senegal, ZIKV strain DAK AR D 41525 obtained from the World Reference Center for Emerging Viruses and Arboviruses (R. Tesh, University of Texas Medical Branch) was used in the studies. The virus was initially amplified, passaged once each in AP61 and C6/36 cells, and then twice in Vero cells. Subsequently, for infection studies in this report, the virus was passaged once in Vero cells (ATCC, CCL-81) and fidelity was verified by sequence analysis [25].

### Mouse Infection Studies

Five-week-old female C57BL/6 mice (*n* = 15/group; Jackson Laboratories) were injected intraperitoneally (IP) with a total of 3.0 mg in three doses (2.0 mg first dose, 0.5 mg sustaining doses) of 5A3 (produced by Leinco Technologies, St. Louis, MO) [26, 27] or phosphate-buffered saline (PBS). The mice were injected on days − 1, + 1, and + 4 PI. In order to saturate the IFNAR-1 receptor, it was determined that a large bolus injection was required initially, *i.e.*, 2.0 mg, followed by subsequent injections to cover the antibody half-life of 5.2 days, [26]. On day 0, mice were infected with 6.4 log_10_ PFU of ZIKV strain DAK AR D 41525 by IP exposure route in a total volume of 200 μl. The animals were evaluated by PET on days 3, 6, and 10 PI. The mean time to death for this mouse model of ZIKV infection is reported to be 9.7 days [24].

Research was conducted under a US Army Medical Research Institute of Infectious Diseases (USAMRIID) IACUC-approved animal research protocol in compliance with the Animal Welfare Act, PHS Policy, and other Federal statutes and regulations relating to animals and experiments involving animals. The facility where this research was conducted is accredited by the Association for Assessment and Accreditation of Laboratory Animal Care, International and adheres to principles stated in the Guide for the Care and Use of Laboratory Animals, National Research Council, 2011.

### qRT-PCR and Plaque Assay to Determine Viral Titers

Tissue and serum samples from mice were processed as previously described [24]. Essentially, mouse serum samples were inactivated using a 3:1 ratio of TRIzol LS Reagent (Thermo Fisher Scientific, Waltham, MA). Tissues were homogenized in 1× minimum essential medium with Earle’s salts and l-glutamine (MEM) with 1 % penicillin/streptomycin and 5 % heat-inactivated fetal bovine serum (FBS-HI) using a gentleMACS dissociator (Miltenyi Biotec, San Diego, CA) followed by centrifugation at 10,000×*g* for 10 min, and the supernatant was stored at − 80 °C until further evaluation. Supernatant was inactivated using a 3:1 ratio of TRIzol LS. Total nucleic acid from all samples was purified using the EZ1 Virus Mini Kit v 2.0 (Qiagen, Valencia, CA) and the EZ1 Advanced XL robot (Qiagen) according to the manufacturer’s recommendations. Samples were eluted in 60 μl. Viral load was determined using a real-time RT-PCR assay specific to the 5′-untranslated region of ZIKV Standard plaque assays were completed as previously described [24] (details are provided in the Electronic Supplementary Material (ESM)).

### PET Radiotracer

[^18^F]DPA-714 was obtained from the Imaging Probe Development Center, NIH, Rockville, MD and was produced by employing slight modifications to procedures already reported [28] and using a commercially available GE TRACERLab FX-N Pro synthesizer [29]. Ready-to-inject, > 99 % radiochemically pure [^18^F]DPA-714 (formulated in physiological saline containing ~ 10 % ethanol) was obtained with 30–40 % (*n* = 12) non-decay-corrected yields and the specific activity at the end of the 70-min radiosynthesis ranged from 48 to 200 GBq/μmol.

### PET/CT Imaging and Data Acquisition

On days 3, 6, and 10 post-infection, mice (*n* = 5/group/day) were bled for determination of viremia by qRT-PCR and then PET/CT scanned. PET/CT scanning was performed using an Inveon preclinical PET/CT system (Siemens Medical Solutions, Knoxville, TN) with a spatial resolution of ~ 1.5 mm full width at half maximum at the center of the field of view. Mice were anesthetized with isoflurane (4 % induction, 2 % maintenance) delivered in oxygen. Once anesthetized, animals were administered ~ 8.0 MBq of [^18^F]DPA-714 in ~ 150 μl volume *via* IV tail vein injection and were allowed to wake in their home cages for a 60-min radiotracer uptake phase. Just prior to scanning, mice were reanesthetized with isoflurane and PET imaging was performed (details are provided in the ESM).

### Image Reconstruction and Data Analysis

All image reconstructions were performed using Siemens’ Inveon Acquisition Workplace v2.0 software package (Siemens Medical Solutions, Knoxville, TN) (details are provided in the ESM). PET images were coregistered to corresponding CT data using VivoQuant v2.5 image processing software (inviCRO, LLC, Boston, MA) and were subsequently coregistered to a 3D mouse brain atlas (included in VivoQuant software package) so that brain [^18^F]DPA-714 biodistribution could be quantified. PET imaging data were reported in terms of percent injected dose per gram of tissue (%ID/g), calculated as a ratio of tissue radioactivity concentration (Bq/g) at time of scan to total injected activity (Bq) at time of scan.

### Histology

Mouse brains were collected at necropsy and fixed by immersion in 10 % neutral buffered formalin for at least 2 days. Brains were subsequently trimmed and processed according to standard protocols [30]. Brain sections were cut at 5 to 6 μM on a rotary microtome, mounted onto glass slides, and stained with hematoxylin and eosin (HE). Histological examination was performed unblinded by a board-certified veterinary pathologist.

### ZIKV RNA *In Situ* Hybridization

*In situ* hybridization was performed using RNAscope® 2.5 HD RED kit according to the manufacturer’s recommendations (Advanced Cell Diagnostics, Hayward, CA) (details are provided in the ESM). The slides were counter-stained with hematoxylin, air dried, and mounted.

### Infrared and Immunofluorescent Imaging

Formalin-fixed, paraffin-embedded mouse brain sections on slides were processed and stained for Iba-1 (details are provided in the ESM). Images were captured on a Zeiss LSM 700 confocal system and processed with Zen 2011 software. A LI-COR-Odyssey CLx (LI-COR Biosciences) scanned sections at 42 μm/pixel resolution. The average intensity of Iba-1 on each slide was obtained from fields-of-interest drawn around each section with the LI-COR-Odyssey analysis software, and two slides per brain were scanned. Negative control staining, for which the primary antibodies were omitted, showed no detectable labeling in immunofluorescence or infrared (IR) imaging.

### Statistical Analysis

Statistical analysis was carried out using SAS/STAT® software, version 9.4 (SAS Institute Inc.). All values are means ± SD, with a testing level (α) of 0.05 and adjusted *p* values as indicated. *p* < 0.05 signifies statistical significance. One-way ANOVA with post-hoc Dunnett’s one-tailed *t* tests were used to compare each treatment group by day with historical control data. One-way ANOVA with post-hoc Tukey-Kramer tests were used to compare between treatment groups by day.

## Results

### Viral Titers in Sera and Brains of ZIKV-Infected Mice

**Fig. 1:**
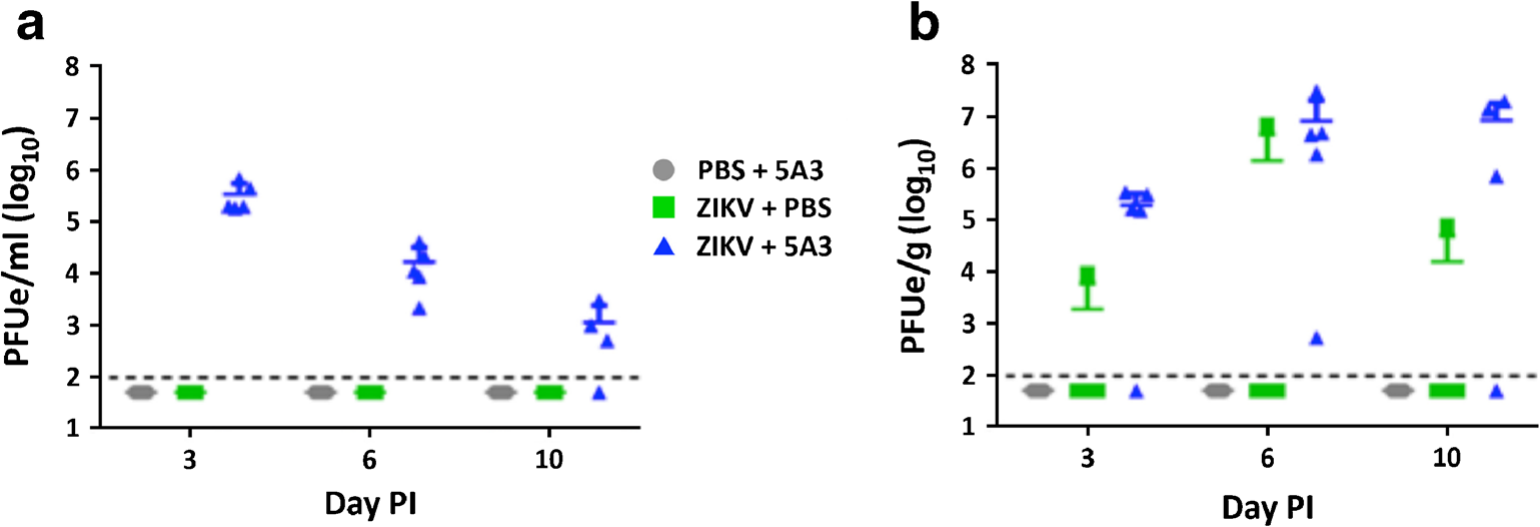
Wild-type mice treated with an IFNAR1-blocking MAb are susceptible to ZIKV. **a** ZIKV RNA in serum. Data are shown as PFU equivalents (*PFUe*) per milliliter after normalization to a standard curve. **b** ZIKV RNA in brain. Data are shown as PFUe per gram after normalization to a standard curve. **a**, **b** *Symbols*, *line*, and *error bars* represent the individual mice, group mean, and standard deviation, respectively. The *dotted line* represents the assay limit of detection.

Prior to PET scanning at days 3, 6, and 10 post-infection, mice designated for imaging from each treatment group were bled and viral titers in sera were determined by qRT-PCR (Fig. 1a). For the PBS + 5A3 and ZIKV + PBS treatment groups, no viremia was detected in any mouse at any time point tested. In the group susceptible to ZIKV infection (ZIKV + 5A3), average viremia was highest (~ 5.5 log_10_ PFUe/ml; *n* = 5) at day 3 PI and decreased at both days 6 (~ 4.2 log_10_ PFUe/ml; *n* = 5) and 10 PI (~ 3.0 log_10_ PFUe/ml; *n* = 4). These data demonstrate a productive infection in the subjects challenged with ZIKV and treated with 5A3 with the exception of one mouse at day 10 PI.

At the conclusion of each PET scan, mice were euthanized and brains were harvested for viral titer determination (Fig. 1b). As expected, the mice in the PBS + 5A3 treatment group showed no detectable viral titers in brain at days 3, 6, or 10 post-infection. In the ZIKV + PBS treatment group, four out of the five mice tested at each time point also showed no detectable viral titers in brain, however, a single mouse at each time point tested (days 3, 6, and 10 post-infection) had viral brain titers of ~ 3.9, ~ 6.8, and ~ 4.9 log_10_ PFUe/g, respectively, suggesting perhaps some level of viral CNS penetration and subsequent clearance with no detectable viremia. In contrast with the decreasing viremia that was measured in the susceptible ZIKV + 5A3 animals, brain titers in this group were shown to increase over time as determined by qRT-PCR from brain homogenates. From day 3 PI to day 10 PI, brain viral titers increased from an average of ~ 5.3 log_10_ PFUe/g at day 3 PI to an average of ~ 6.95 log_10_ PFUe/g in the remaining living mice (*n* = 4/5) by day 10 PI. It should be noted, however, that at days 3 and 10 post-infection, a single mouse at each time point, respectively, had brain viral titers below the limit of detection for our assay. To confirm the presence of infectious virus in the brain, qRT-PCR titers were verified by conventional plaque assay. Altogether, these data are consistent with our previous findings [24] and those of others [20–23] and demonstrate a substantial increase in ZIKA neuroinvasion when IFN-I signaling is blocked.

### [^18^F]DPA-714 Uptake in Brains of Control and ZIKV-Infected Mice

**Fig. 2:**
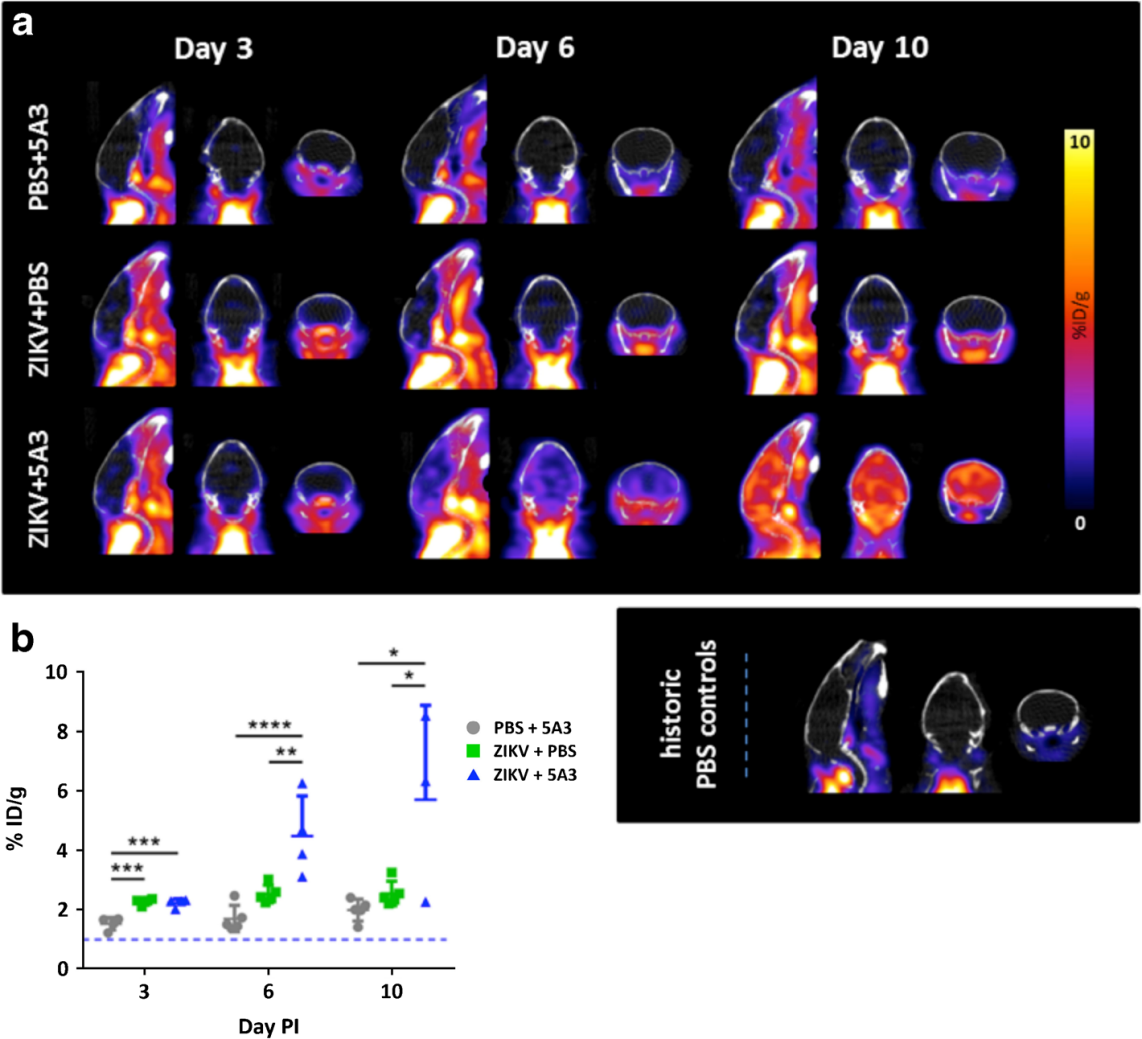
*In vivo* PET imaging can detect ZIKV-related neuroinflammation as early as day 3 post-infection (*PI*). Mice were separated into three treatment groups, PBS + 5A3, ZIKV + PBS, and ZIKV + 5A3 and were IV injected with [^18^F]DPA-714 at 3, 6, and 10 days PI to quantitatively assess brain neuroinflammation in terms of percent injected dose per gram tissue (%ID/g). **a** Representative PET/CT mouse brain images (sagittal, coronal, and transverse, from left to right) for each treatment group at days 3, 6, and 10 PI. Representative historic PBS control mouse brain images at *bottom right*. **b** *Symbols*, *line*, and *error bars* represent the individual mice, group mean, and standard deviation, respectively. The *dotted line* represents the mean [^18^F]DPA-714 binding value in brains from PBS-treated mice (~ 1.06 ± 0.21 %ID/g). *p < 0.05; **p < 0.01; ***p < 0.005; ****p < 0.001; one-way analysis of variance (ANOVA).

To evaluate the ability of *in vivo* PET imaging to detect ZIKV-induced neuroinflammation in mouse brain, we used [^18^F]DPA-714, a PET radiotracer that is specific for TSPO [11], a biochemical marker of neuroinflammation that is highly upregulated in activated microglia, CNS macrophages, and reactive astrocytes [12–19]. By as early as day 3 PI, ZIKV-infected mice treated with MAb-5A3 showed a modest but significant (approximately twofold) increase in mean whole-brain [^18^F]DPA-714 binding (2.21 ± 0.14 %ID/g; *n* = 5) compared with historic PBS controls (Fig. 2a, b). By days 6 and 10 post-infection, the increase in mean whole-brain [^18^F]DPA-714 binding in ZIKV + 5A3 mice was approximately four- (4.48 ± 1.35 %ID/g; *n* = 5) and sixfold (5.70 ± 3.19 %ID/g; *n* = 3), respectively, compared with historic PBS controls. The changes in whole-brain [^18^F]DPA-714 binding was highly correlated with brain viral titer, *i.e.*, *r* = 0.716. Based on brain subregion analysis, all brain regions evaluated (olfactory bulb, striatum, hippocampus, thalamus, hypothalamus, cortex, and cerebellum) were increased similar to the whole brain (details are provided in Table 1 of the ESM). Of note, one of the three mice from the ZIKV + 5A3 group at day 10 PI had [^18^F]DPA-714 binding (2.25 %ID/g) similar to the ZIKV + PBS (2.53 ± 0.41 %ID/g, *n* = 5) and PBS + 5A3 (1.97 ± 0.37 %ID/g, *n* = 5) control groups at day 10 PI, which reduced the mean whole-brain [^18^F]DPA-714 binding for this group. Moreover, viral RNA was not detected in the brain of this mouse at day 10 PI (Fig. 1b). This subject was not excluded from our analyses because viremia was not assessed in this subject at day 3 and/or day 6 PI allowing for the possibility that this subject was infected and cleared the virus prior to CNS penetration, *i.e.*, it recovered from the neuroinflammatory disease prior to day 10 or it never became infected.

ZIKV alone (ZIKV + PBS) showed a similar modest (2.25 ± 0.11 %ID/g; *n* = 5) but significant increase in mean whole-brain [^18^F]DPA-714 binding at day 3 PI relative to historic PBS controls. Mean whole-brain [^18^F]DPA-714 binding in ZIKV + PBS remained at modest levels at day 6 PI (2.51 ± 0.30 %ID/g, *n* = 5) and returned to the level of historic PBS controls by day 10 PI suggesting that ZIKV infection in the presence of IFN signaling is sufficient to produce a mild yet transient neuroinflammatory effect measureable by [^18^F]DPA-714 PET imaging. Importantly, 5A3 treatment in the absence of ZIKV had no detectable effect on mean whole-brain [^18^F]DPA-714 uptake and shared a similar tracer uptake profile as the historic PBS control mice at all three time points tested. These results demonstrate for the first time the ability of [^18^F]DPA-714 PET imaging to detect and quantify ZIKV-related neuroinflammation disseminated throughout the brains of infected mice.

### Histopathology Findings

**Fig. 3:**
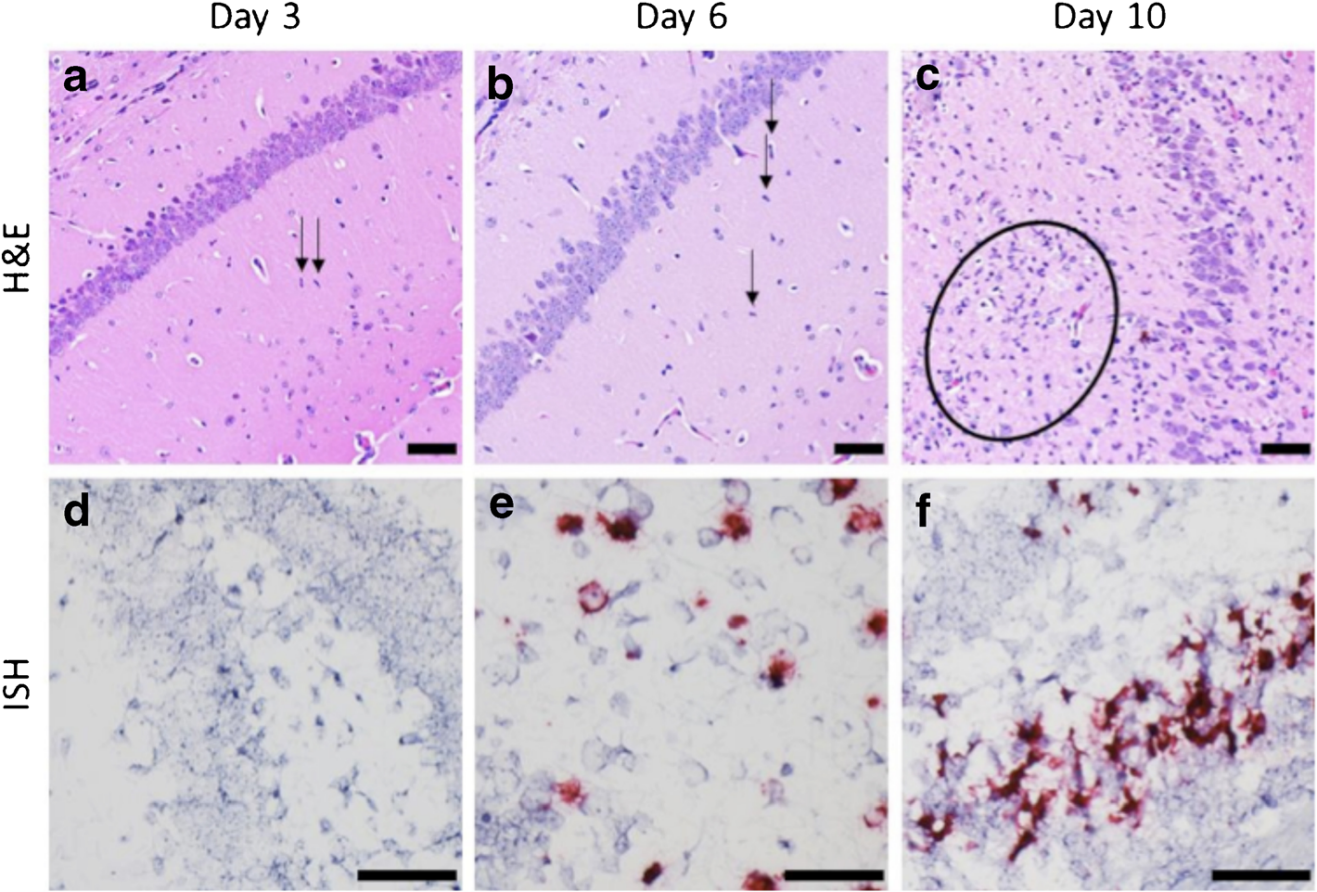
Microgliosis and ZIKV RNA in ZIKV-infected, wild-type mouse brains treated with MAb-5A3. **a**–**c** Brain sections were examined by hematoxylin and eosin, which showed **a** minimal microgliosis at day 3 PI (*arrows*), **b** minimal-to-mild microgliosis at day 6 PI (*arrows*), and **c** moderate microgliosis at day 10 PI (*circle*). *Scale bar* represents 100 μm. **d**–**f** Representative images of ISH staining of ZIKV RNA in the brains of MAb-5A3-treated, ZIKV-infected mice. ZIKV RNA is absent at day 3 PI (**d)**, present in cerebral cortex at day 6 PI (**e**), and in hippocampus plus cerebral cortex at day 10 PI (**f**). *Scale bar* represents 50 μm.

To confirm the *in vivo* findings of ZIKV-induced neuroinflammation as determined by [^18^F]DPA-714 PET imaging, brain sections from the PET imaged mice were assessed for pathology, *in situ* hybridization (ISH) to detect viral RNA, and IR and immunofluorescent detection of Iba-1, a microglia/macrophage-specific calcium-binding protein that is highly upregulated during neuroinflammation [31]. Consistent with our imaging results, brains from the susceptible ZIKV + 5A3 treatment group exhibited histopathological lesions consistent with encephalitis with minimal microgliosis beginning as early as day 3 PI (Fig. 3a). By days 6 and 10 PI, microgliosis was more pronounced, with the presence of mononuclear cell perivascular cuffs peaking at day 6 PI and waning by day 10 PI (Fig. 3b, c). Other histological findings consistent with encephalitis include minimal necrotic cellular debris scattered throughout the parenchyma (days 6 and 10 PI) and neuronal degeneration and necrosis observed in the cerebrum as early as day 6 PI and becoming more pronounced and widespread on day 10 PI (cerebrum, hippocampus, and thalamus). Additionally, variable minimal perivascular edema and hemorrhage was present on both days 6 and 10 PI. Moreover, ZIKV RNA was detected by ISH in the cerebral cortex on days 6 and 10 PI and in the hippocampus on day 10 PI in mice administered ZIKV + 5A3 (Fig. 3e, f). No ZIKV RNA was detected on day 3 PI in mice administered ZIKV + 5A3 (Fig. 3d). Mice administered ZIKV + PBS exhibited no signs suggestive of ZIKV infection on day 3 or 6 PI; however, 1/5 animals exhibited minimal lesions consistent with encephalitis (perivascular cuffing, microgliosis) on day 10 PI that may be attributable to ZIKV infection, though no viral RNA was detected in this animal by ISH. As expected, animals administered 5A3 + PBS exhibited no CNS lesions suggestive of ZIKV infection and no viral RNA was detected by ISH at any of the time points tested.

**Fig. 4:**
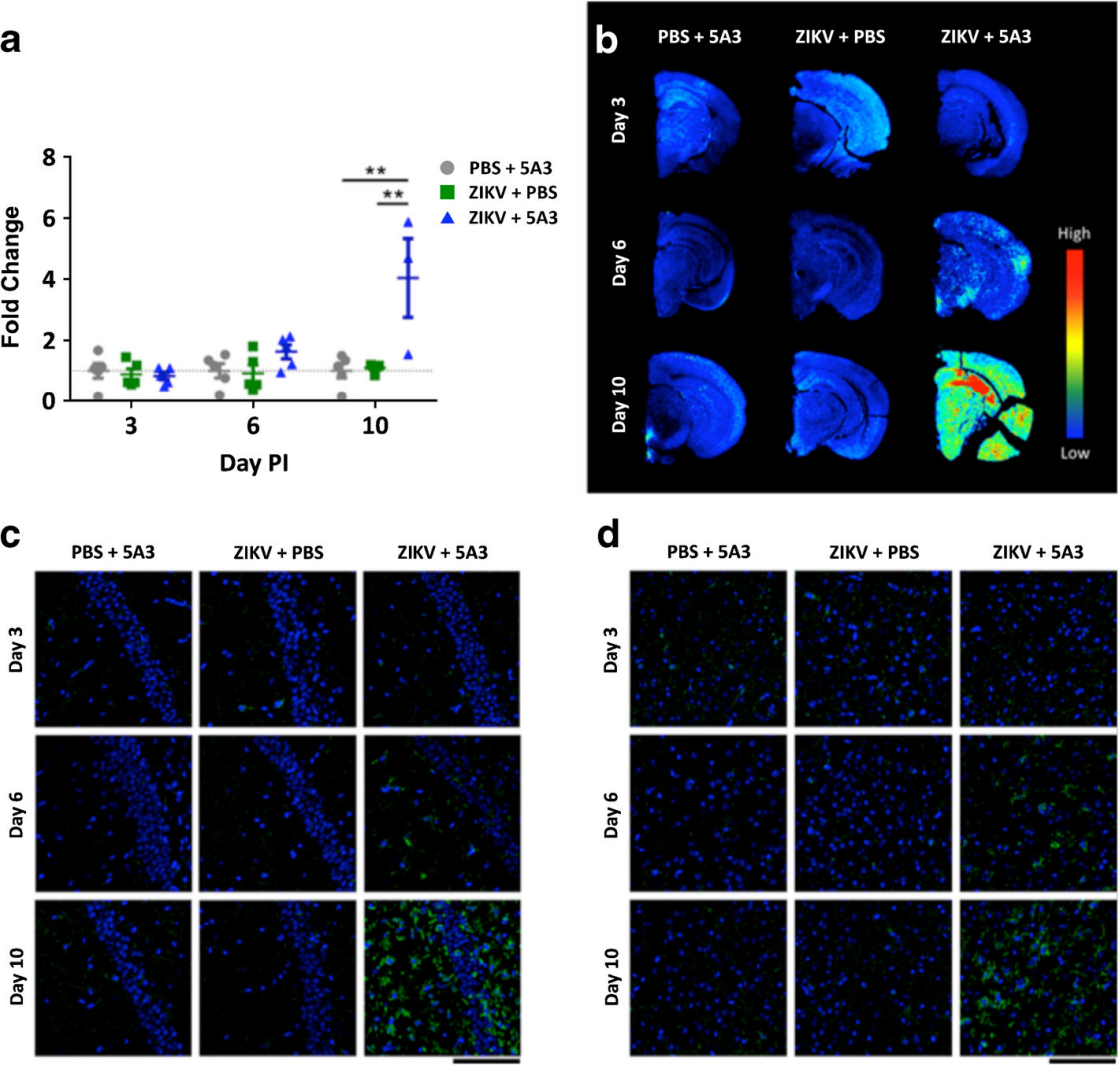
Iba-1 is increased in the brains of ZIKV-infected wild-type mice treated with an IFNAR1-blocking MAb. Brain sections analyzed for Iba-1 expression by infrared (IR) and immunofluorescent imaging for all three treatment groups, PBS + 5A3, ZIKV + PBS, and ZIKV + 5A3. **a** By day 10 PI, the average IR intensity was increased approximately fourfold relative to PBS + 5A3 controls (**p < 0.01, one-way analysis of variance (ANOVA)). *Symbols*, *line*, and *error bars* represent the individual mice, group mean, and standard deviation, respectively. **b** Representative images show increased Iba-1 IR signal in brain sections from ZIKV + 5A3 mice compared with PBS control groups. **c** Representative CA1 hippocampal and **d** cortical sections showing immunofluorescent labeling of Iba-1 (*green*) and nuclei (*blue*) in PET imaging mice. *Scale bar* represents 200 μm.

In terms of Iba-1 expression, no statistical difference was observed by IR imaging at days 3 and 6 PI for any of the treatment groups tested, PBS + 5A3, ZIKV + PBS, or ZIKV + 5A3 (Fig. 4a, b, first two rows). At day 6 PI, however, whole-brain Iba-1 expression in the susceptible ZIKV + 5A3 group is modestly, though not significantly, increased. By day 10 PI, and in agreement with the [^18^F]DPA-714 findings, Iba-1 expression was significantly increased (approximately fourfold) in the ZIKV + 5A3 group compared with either of the control groups (Fig. 4a, b, bottom row). These findings were confirmed by immunofluorescent labeling of Iba-1 in these brains (Fig. 4c, d). Importantly, the single animal in the ZIKV + 5A3 treatment group at day 10 post-infection that showed no appreciable Iba-1 labeling by IR imaging was the same animal that at day 10 PI showed no detectable virus in the brain (Fig. 1b) and no significant whole-brain [^18^F]DPA-714 binding over controls (Fig. 2a, b). These data are consistent with and serve to confirm our *in vivo* PET imaging results. These results also demonstrate the ability of *in vivo* [^18^F]DPA-714 PET imaging to provide a more sensitive measure of ZIKV-induced neuroinflammation than Iba-1 IR or immunofluorescent imaging.

## Discussion

Here, we demonstrate that [^18^F]DPA-714 PET imaging is a sensitive method capable of identifying and quantifying ZIKV-induced neuroinflammation in a previously described mouse model of ZIKV neurological disease. In this model, C57BL/6 mice with a transiently suppressed IFN-I response by treatment with MAb-5A3 are highly susceptible to peripheral ZIKV challenge and [^18^F]DPA-714 PET imaging was able to detect significant neuroinflammation at day 3 PI compared with uninfected, 5A3-treated mice. Neuroinflammation continues to increase at days 6 and 10 PI in the 5A3-treated, ZIKV-infected mice. In addition, [^18^F]DPA-714 PET imaging was able to detect modest, yet significant neuroinflammation in immunocompetent mice exposed to ZIKV and treated with PBS at day 3 PI demonstrating the induction of neuroinflammation in the absence of 5A3 treatment. Neuroinflammation in this group begins to wane by day 6 PI and is indistinguishable from historic PBS controls by day 10 PI. This transient, low-level neuroinflammation, in combination with the lack of viremia and virus in the brain, may result from the innate and adaptive immune responses against ZIKV infection. Taken together, these results indicate that neuroinflammation plays an important role in the pathogenesis of ZIKV infection and that [^18^F]DPA-714 PET imaging can sensitively detect neuroinflammation in both immune-compromised and immunocompetent mice exposed to ZIKV.

Previous studies have shown that ZIKV infection in animal models is associated with the activation of immune responses, cellular infiltration, inflammation and neural damage in the CNS [24, 32]. It is interesting to note that in our study, significant neuroinflammation was detected throughout the brains of mice despite limited detection of viral RNA by ISH (Fig. 3) and qRT-PCR (Fig. 1b). But surprisingly, a strong correlation between brain viral titer and [^18^F]DPA-714 binding was noted. These findings suggest that the host inflammatory response is associated with the ZIKV-induced neuropathogenesis which is not solely caused by virus-mediated damage. This is consistent with a recent study in *Stat2* ^*−/−*^ mice demonstrating that ZIKV pathogenicity associates with the degree of inflammatory immune response in the CNS of infected mice and does not correlate with viral RNA levels [33]. The immune response plays a critical role in the outcome of alphavirus-induced CNS disease as well. Increasing evidence suggest that fatal alphavirus encephaloymyelitis is mediated by the immune response to virus infection rather than direct virus replication (reviewed in [34, 35]). Collectively, these results suggest that the use of [^18^F]DPA-714 PET to measure ZIKV-induced neuroinflammation may provide a more accurate quantification of ZIKV pathology than viral titer measurements alone. Therefore, [^18^F]DPA-714 PET imaging may not only more accurately monitor disease progression during ZIKV infection, but it may also serve as a dynamic measure for assessment of drug efficacy.

The results of the current study are also consistent with previous studies that used AG129 mice which lack interferon α/β and γ receptors (IFN-α/β and IFN-γ). ZIKV is lethal in AG129 mice, but significant neuropathology is observed prior to death [20–23]. Neurodegeneration, as evidenced by necrotic cellular debris, nuclear fragments, hyper-eosinophilic cytoplasm, and pycnotic nuclei is observed in AG129 mice infected with ZIKV. Inflammatory cells such as lymphocytes, monocyte-macrophages, neutrophils and T-cells are also seen in the parenchyma of the brain [20, 21]. In AG129 mice as well as in neonatal C57BL/6 mice infected with ZIKV, genes associated with inflammation and cellular infiltration are significantly upregulated in the brain [22]. The gene expression in AG129 mice was consistent with inflammatory infiltrates, activated microglia and astrocytes, while in neonatal C57BL/6 mice with an intact immune system, a phenotype associated with a significant T cell response was noted [22]. Thus, the results of the current study together with previously published work demonstrate the importance of the IFN signaling pathway in the resolution of ZIKV infection, and that neuroinflammation is a key component of ZIKV infection that if left unresolved, leads to death (*e.g.*, ZIKV + 5A3 group *versus* ZIKV + PBS).

[^18^F]DPA-714 PET imaging also proved to be more sensitive for the detection of small changes in neuroinflammation during ZIKV infection relative to classical immunohistochemical methods. [^18^F]DPA-714 binding was significantly increased at days 3, 6, and 10 PI in 5A3-treated, ZIKV-infected mice compared with uninfected, 5A3-treated mice, while significant Iba-1 staining was only noted at day 10 PI. In addition, [^18^F]DPA-714 binding was significantly increased at day 3 PI in the ZIKV + PBS treatment group while the change was undetected by Iba-1 staining. These findings demonstrate the ability of PET imaging to detect pathology/neuroinflammation earlier and throughout the course of ZIKV infection with greater sensitivity than classical methods, and that the approach can be used to dynamically study early pathogenic events associated with ZIKV and potentially other neurotropic viral infections. Moreover, it may be possible to define distinct disease stages or mechanisms associated with the pathogenesis of ZIKV infection or other neurotropic viruses. For example, PET imaging could identify routes of neuroinvasion or mechanisms of blood-brain barrier permeability and then quickly assess this route or mechanism in related or different neurotropic viruses, thus allowing this technology to expedite the process of expanding an efficacious therapeutic for one virus, to potentially others. In future work, it would be interesting to evaluate the same animal over time post-infection to better define the disease stages and more dynamically assess the effect of ZIKV on generation of neuropathology. However, with a dynamic imaging approach correlations between the imaging endpoints and classical histologic measures and tissue/serum viral levels would be lost unless separate cohorts were also evaluated.

## Conclusions

Our findings demonstrate that global neuroinflammation plays a significant role in the progression of ZIKV infection in an immunocompromised mouse model and that [^18^F]DPA-714 PET imaging is a sensitive tool relative to histology for the detection of neuroinflammation. [^18^F]DPA-714 PET imaging may be useful in dynamically characterizing the pathology associated with neurotropic viruses and the evaluation of therapeutics being developed for treatment of infectious diseases.

## Electronic Supplementary Material

ESM 1 (PDF 155 kb).
