# Supplementary material for: [18F]DPA-714 PET Imaging Reveals Global Neuroinflammation in Zika Virus-Infected Mice
Source: Mol Imaging Biol. 2017 Sep 12;20(2):275–83. doi: 10.1007/s11307-017-1118-2 (PMC5862915; doi:10.1007/s11307-017-1118-2)
Supplement: Supplementary file 1 — (PDF 155 kb). [file 11307_2017_1118_MOESM1_ESM.pdf]

Electronic Supplementary Material

[^{18}F]DPA-714 PET Imaging Reveals Global Neuroinflammation in Zika Virus-Infected Mice

Journal: Molecular Imaging and Biology

Kyle Kuszpit[1†], Bradley S. Hollidge[2†], Xiankun Zeng[3], Robert G. Stafford[1], Sharon Daye[3], Xiang Zhang[4], Falguni Basuli[4], Joseph W. Golden[2], Rolf E. Swenson[4], Darci R. Smith[2†*], Thomas M. Bocan[1†*]

Affiliations:

[1]Molecular and Translational Sciences Division, [2]Virology Division, [3]Pathology Division, U.S. Army Medical Research Institute of Infectious Diseases, 1425 Porter St., Ft. Detrick, MD., 21702 USA

[4]Imaging Probe Development Center, National Heart, Lung, and Blood Institute, National Institutes of Health, 9800 Medical Center Drive, Bldg. B., #2034
Bethesda, MD., 20892 USA

†These authors contributed equally to this work.

*Corresponding authors: Thomas Bocan; thomas.m.bocan.ctr@mail.mil; (301) 619-2647 and Darci Smith; darci.r.smith.ctr@mail.mil; (301) 619-4125

Material and Methods

qRT-PCR Assay to Determine Viral Titer in Sera and Brain

Viral load was determined using a real-time RT-PCR assay specific to the 5'-untranslated region of ZIKV. Specific amplification detection was accomplished using a forward primer (5'-GARTCAGACTGCGACAGTTCGA), reverse primer (5'-CCAAATCCAAATTAAACCTGTTGA), and probe (5'-ACTGTTGTTAGCTCTCGC–MGBNFQ). A standard curve was generated using serial dilutions of the challenge virus where the PFU/ml titers were determined by plaque assay. Five µl of extracted nucleic acid were run in triplicate on the LightCycler 480 (Roche Diagnostics, Inc., Indianapolis, IN) using SuperScript One-Step RT-PCR (Thermo Fisher Scientific), and samples were considered negative if the cycle of quantification (Cq) was greater than 40 cycles. This Cq cutoff value was selected because greater than 40 cycles is outside of the linear dynamic range of the real-time PCR assay. The virus titers were calculated using the standard curve and the LightCycler 480 software, and the final PFU equivalents/ml (PFUe/ml) calculations were determined based on the sample input volumes and the upfront sample dilutions.

Virus Titration and Plaque Assay

Vero cells were plated at $3x10^5$ cells/well in a six-well plate and incubated overnight at 37°C, 5% CO_2. Serial dilutions of samples were made in 1X MEM with 1% penicillin/streptomycin and 5% heat-inactivated FBS. Uninfected-control and serially diluted samples were incubated with the Vero cells for one hour at 37°C, 5% CO_2 for virus adsorption. The inoculum was removed and a 1:1 mixture of 0.8% (w/v) Seaplaque agarose and 2X Basal

Medium Eagle with Earle's Salts (EBME) solution containing 2X EBME, 10% FBS-HI, 2% penicillin/streptomycin, 50 µg/ml gentamicin, and 2.5 µg/ml Fungizone/Amphotericin B was added. After addition, the 0.4% Seaplaque agarose/2X EBME overlay was incubated at room temperature for 30 min to allow the overlay to solidify. Vero cells were incubated with the overlay at 37°C, 5% CO_2 for five days before the overlay was removed. Cells were fixed and plaques were visualized by a 20 min addition of 10% formalin with 50% Crystal Violet solution followed by a wash with water.

PET/CT Imaging and Data Acquisition

Just prior to PET scanning, mice were reanesthetized with isoflurane and placed in the scanner positioned in the center of the PET field of view. A single, 15 min static PET scan was initiated followed immediately by CT scanning (80 kV, 500 µA, 98 µm, 360º rotation in 220 steps). During all imaging procedures, animal respiration rate and body temperature were monitored and maintained using an M2M-BioVet™ small animal physiological monitoring system (M2M imaging, Cleveland, OH). When scanning was completed, mice were euthanized via CO_2 asphyxiation and brains were removed and preserved for infrared (IR), immunohistological and tissue viral titer analysis. PET and CT imaging data were reconstructed and [^{18}F]DPA-714 biodistribution was quantified and evaluated for differences between treatment groups at days 3, 6, and 10 post-treatment and compared to historic PBS-administered controls. The historic PBS-administered controls consisted of 6 female C57BL/6 mice (Jackson Laboratories) of approximately five weeks of age that were evaluated by [^{18}F]DPA-714 imaging in a manner consistent with that reported here for the ZIKV infected mice.

Image Reconstruction and Data Analysis

All image reconstructions were performed using Siemens' Inveon Acquisition Workplace v2.0 software package (Siemens Medical Solutions, Knoxville, TN). Raw CT attenuated data were reconstructed and the values normalized to air and water to produce normalized Hounsfield units (HU). The CT data were reconstructed using a Feldkamp reconstruction algorithm with a Shepp-Logan reconstruction filter, slight noise reduction, and beam hardening correction applied. PET images were generated using a 3-dimensional ordered subset expectation maximization (OSEM) with three iterations and 12 subsets followed by a maximum a posteriori (MAP) algorithm with 18 iterations. Scatter and attenuation correction were performed and a decay correction for F-18 was applied. With a zoom factor of 1.0 and a 128 x 128 x 159 matrix, a final voxel dimension of 0.78 x 0.78 x 0.80 mm was obtained._PET images were coregistered to corresponding CT data using VivoQuant v2.5 image processing software (inviCRO, LLC, Boston, MA) and were subsequently coregistered to a 3D mouse brain atlas (included in VivoQuant software package) so that brain [^{18}F]DPA-714 biodistribution could be quantified. PET imaging data were reported in terms of percent injected dose per gram of tissue (%ID/g), calculated as a ratio of tissue radioactivity concentration (Bq/g) at time of scan to total injected activity (Bq) at time of scan.

ZIKV RNA In situ Hybridization (ISH)

In situ hybridization was performed using RNAscope® 2.5 HD RED kit according to the manufacturer's recommendations (Advanced Cell Diagnostics, Hayward, CA) (Details are provided in ESM). Briefly, 20 ZZ probes set targeting the 1550-2456 fragment of the ZIKV polyprotein gene with Gene bank accession KJ776791.1 were synthesized. After

deparaffinization and peroxidase blocking, the sections were heated in antigen retrieval buffer and then were digested by proteinase. The sections were covered with ISH probes and incubated at 40°C in a hybridization oven for 2 h. They were rinsed and the ISH signal was amplified by applying Pre-amplifier and Amplifier conjugated with horse radish peroxidase (HRP). A red substrate-chromogen solution was applied for 10 min at room temperature. The slides were further stained with hematoxylin, air dried, and mounted.

Infrared (IR) and Immunofluorescent Imaging

Formalin-fixed, paraffin-embedded mouse brain sections on slides were processed and stained for Iba-1 deparaffinized in xylene and rehydrated through graded ethanol (100%, 95%, 90%, and 70%). Antigen was retrieved by citric acid-based antigen unmasking solution (Vector Laboratories) during 10 min at 95-100ºC. After three washes with PBS (pH 7.4), the sections were blocked with 10% normal donkey serum in PBS-tween (0.1%; PBS-T) for one hour at room temperature. The sections were incubated with primary antibody, goat anti-Iba-1 (3 µl/ml; Novus Biologicals, Littleton, CO), diluted in 10% normal donkey serum in PBS-T overnight at 4°C. After washing in PBS-T (3x5 min), the sections were incubated overnight at 4°C with secondary antibodies diluted in 10% normal donkey serum in PBS-T. For standard immunofluorescence, the secondary antibodies were donkey anti-goat Alexa Fluor 594 (1:400; Invitrogen; pseudocolored green). The nuclei were stained with Hoecht's. For IR analysis, the secondary antibody was donkey anti-goat IRDye 680RD (1:1500; LI-COR Biosciences). The sections were subsequently washed in PBS-T (3 x 10 min), PBS (3 x 5 min), and water (2 x 5 min). For immunofluorescence, the sections were cover slipped with Fluoromount-G (Southern Biotech, Birmingham, AL). Images were captured on a Zeiss LSM 700 confocal system and

processed with Zen 2011 software. Sections for IR analysis were air-dried overnight. A LI-COR-Odyssey CLx (LI-COR Biosciences) scanned sections at 42 μm/pixel resolution. The average intensity of Iba-1 on each slide was obtained from fields-of-interest drawn around each section with the LI-COR-Odyssey analysis software and two slides per brain were scanned. Negative control staining, for which the primary antibodies were omitted, showed no detectable labeling in immunofluorescence or IR imaging.

Table 1: Sub-region Analysis of [^{18}F]DPA714 Binding in the Brain

Brain Region	Treatment	Day 3 Post-Infection	Day 6 Post-infection	Day 10 Post-infection
Olfactory Bulbs	PBS Control	1.212 ± 0.106	-	-
	PBS+5A3	1.676 ± 0.339	1.898 ± 0.820	2.251 ± 0.686
	ZIKV+PBS	2.671 ± 0.177	2.943 ± 0.426	2.830 ± 0.316
	ZIKV+5A3	2.609 ± 0.190	5.121 ± 1.647	5.640 ± 3.180
Cortex	PBS Control	0.919 ± 0.088	-	-
	PBS+5A3	1.344 ± 0.238	1.449 ± 0.348	1.644 ± 0.319
	ZIKV+PBS	1.944 ± 0.089	2.094 ± 0.214	2.125 ± 0.450
	ZIKV+5A3	1.851 ± 0.127	3.946 ± 0.982	5.095 ± 3.002
Hypothalamus	PBS Control	1.292 ± 0.215	-	-
	PBS+5A3	1.702 ± 0.474	2.167 ± 0.740	2.560 ± 0.749
	ZIKV+PBS	3.159 ± 0.291	3.856 ± 0.620	3.710 ± 0.497
	ZIKV+5A3	2.846 ± 0.260	5.217 ± 1.262	6.364 ± 3.045
Thalamus	PBS Control	0.801 ± 0.075	-	-
	PBS+5A3	1.147 ± 0.269	1.044 ± 0.307	1.278 ± 0.260
	ZIKV+PBS	1.389 ± 0.104	1.506 ± 0.202	1.711 ± 0.513
	ZIKV+5A3	1.448 ± 0.043	3.492 ± 1.531	5.540 ± 3.907
Hippocampus	PBS Control	0.955 ± 0.086	-	-
	PBS+5A3	1.504 ± 0.307	1.464 ± 0.423	1.817 ± 0.345
	ZIKV+PBS	2.083 ± 0.168	2.352 ± 0.231	2.398 ± 0.453
	ZIKV+5A3	2.144 ± 0.101	4.271 ± 1.326	6.047 ± 3.581
Striatum	PBS Control	0.823 ± 0.087	-	-
	PBS+5A3	1.190 ± 0.247	1.282 ± 0.305	1.504 ± 0.308
	ZIKV+PBS	1.804 ± 0.102	1.996 ± 0.252	2.067 ± 0.501
	ZIKV+5A3	1.761 ± 0.102	4.215 ± 1.785	5.589 ± 3.489
Cerebellum	PBS Control	1.440 ± 0.163	-	-
	PBS+5A3	2.461 ± 0.480	2.427 ± 0.615	2.862 ± 0.296
	ZIKV+PBS	3.137 ± 0.198	3.458 ± 0.356	3.434 ± 0.411
	ZIKV+5A3	3.128 ± 0.255	5.222 ± 1.317	6.196 ± 2.554

The data are represented as Mean ± Standard Deviation in %ID/gm tissue.
